# 

**Published:** 1857-11

**Authors:** 


					﻿PUBLISHED MONTHLY, AT S3 PER ANNUM IN ADVANCE.
X
&
5
>-
o
s
©
3
*S
ft
■
6
fa
ft
©
a
T
o
-
2
cr
5
g
•
6
&
a
X
S
©
©
©
co
x
3
g
©
©
©
>
©
t*
a
©
A
©
*
a
©
O
to
H
e
3
g
©
©
fa
©
•c
a
P
le
»
©
ft-
A. T L TV 1ST T TV
MEDICAL ISO Ol|
■ J-fi I 11 1 L ,
EDITED BY
JOSEPH P. LOGAN, M. D.,
i’ROFM E OF PlITSlOLOGT ARB GkXERAL PAinOLOGT IX TDK All AX."A Y DICAL COI '.EOF.
AND
W. F. WESTMORELAND, M. D.,
I*HOIessQP. OF THE Pbixciplks axd Practice of Surgery is tiie Ailaxta Medical Cmuci,
/ - -• - ----- - - - . _ .
Pax et scientia, sed veritas sine timore.
Vol. iti. NOVEMBER, 1857. No. in.
ATLANTA, GEORGIA:
G. I*. EDDY & CO., PUBLISHERS AND PROPRIETORS.
1857,
EACH NUMBER CONTAINS SIXTY-FOUR PAGES.
a*
X
• s
x
X
a
©
o
a
=
X
2
©
©
a
©
5.
s’
ft
X
a
©
©*
5
s
r
C-
2
w
z
>
•M
ST
►-
©
QD
ft
a
?
©
B
©
©
X
				

